# Seed morphology characteristics in relation to seed loss by water erosion in the Loess Plateau

**DOI:** 10.1186/2193-1801-2-S1-S9

**Published:** 2013-12-11

**Authors:** Juying Jiao, Luyan Han, Yanfeng Jia, Dong Lei, Ning Wang, Linyu Li

**Affiliations:** Institute of Soil and Water Conservation, Northwest A&F University, Yangling, Shaanxi 712100 China; Institute of Soil and Water Conservation, Chinese Academy of Sciences and Ministry of Water Resources, Yangling, Shaanxi 712100 China

**Keywords:** seed loss, seed displacement, seed morphology, water erosion, simulation rainfall experiment

## Abstract

The role of water erosion on seed loss and on plant establishment and distribution is unknown on the Chinese Loess Plateau, which suffers serious soil erosion. The seed susceptibility of 16 local species to removal by water erosion from loess slopes was determined by rainfall simulation experiments. The experiments were performed on slopes with gradients of 10°, 15°, 20° and 25° for a 60-min duration at an intensity of 50 mm/h, 100 mm/h and 150 mm/h, respectively. The total seed removal rate obviously increased with rainfall intensity but did not obviously change with slope gradient, and the responses were varied among the species. The morphological characteristics affecting seed loss of the various species are quite different. Our experiments showed that the seed removal from some species are mainly due to seed weight, some species are mainly affected by seed shape, some are affected by appendage, some by surface structure, some by the comprehensive effects of the different morphological characteristics, while seeds having mucilage secretion are not easily moved by water erosion. We argued that the seed removal during water erosion can clearly effect seed redistribution and deposition, and consequently, species composition and vegetation spatial distribution.

## Introduction

In soil erosion environments, the overland flow and sediment transport also can carry away the seeds that arrive at the soil surface and those that remain in the soil [1–3]. The seed loss caused by water erosion may cause post-dispersal of seed, alter the primary seed dispersal and deposition pattern [4], lead to seed redistribution, and determine seed spatial colonization, survival and seedling establishment [5–7]. The influence of the spatial distribution of seedling renewal, therefore, plays an important role in vegetation restoration and succession [4, 8]. Therefore, seed loss may be one of the main factors hindering vegetation recover, and it may explain the scarcity or the absence of vegetation on badland slopes [3, 9].

Seed removal caused by water erosion has deservedly been given much attention since 1995. The following have been investigated: seed loss by water erosion [3, 9], the influence of seed size and shape [10], and slope angle [11] on seed losses in degraded Mediterranean environments (southeastern Spain); the contributions to seed loss control of plant canopy in northern Ethiopia [4], bioengineering works [12], and vegetation cover and hoof prints [13, 14] in the French Alps. The above previous study in the degraded Mediterranean environments showed that the total seed losses were less than 10% for all replicates and there was no species that lost more than 25% of its seeds in any replication [9]; and the amount of seed loss was very low on 22-55° slopes with only 4% of the seeds eroded [11]. Thus, it has been concluded that seed removal by water erosion is not the key factor in the lack of vegetation on badlands in southeastern Spain, but factors related to seed germination and seedling survival may play an important role [3, 11].

However, the above studies in southeastern Spain were just conducted in the rainfall intensity of 55 mm/h corresponds to the smallest rainstorm intensities that are able to generate runoff and water circulation in watersheds in SE Spain, which has a return period of 10 years [9]. When the heavier rainstorms occurred, the seed losses by water erosion would show different responses. While in Northern Ethiopia, the seed loss was 32.5% at a simulated rainfall with a maximum intensity of 120 mm/h for a 10 min duration [4]. Moreover, for the 83 species used in the experiment [10], greater erosion rates were found for species *E. multiflora* (93%), *S. sediforme* (68%) and *P. pinnatifidum* (56%). It was also argued that the seed susceptibility to be removed by water erosion has the potential to affect plant communities of semi-arid Mediterranean slopes [15].

Species composition changes in different regions with various seed morphological characteristics, and the seeds in the experiments above couldn't include all the seed morphology features in other regions which undergo severe soil erosion. Different species has different response to seed removal caused by water erosion due to their specific seed morphological characteristics. Seed mass is the main characteristic explaining seed susceptibility to removal by water erosion and that seed shape becomes important only after seeds reach a threshold mass [9, 10]. And the susceptibility of a seed to be removed by erosion can be modified by the presence of seed appendages (hairs, wings, awns) or by the seed ability to segregate mucilage in contact with water [9].

The Chinese Loess Plateau is well-known for its severe soil erosion and degraded ecosystem, and the role of water erosion in the seed loss and then in the successive plant establishment and distribution is little known. Therefore, the aim of this research is to experimentally investigate the relationships between seed morphological characteristics and seed susceptibility to be removed by water erosion under different rainfall intensities and slope gradients by using the seeds of species and loess soil in the Loess Plateau region, and detect the effect of seed loss during water erosion on vegetation development and distribution combining with field survey of vegetation.

## Materials and methods

### Seeds

Seeds from the following 16 species were used in the experiments: *Heteropappus altaicus* (HA), *Bidens bipinnata* (BB), *Dracocephalum moldavica* (DM), *Clematis florida* (CF), *Lespedeza davurica* (LD), *Periploca sepium* (PS), *Daucus carota* (DC), *Bothriochloa ischaemun* (BI), *Robinia psendoacacia* (RP), *Rubia cordifolia* (RC), *Cotoneaster multiflorus* (CM), *Patrinia heterophylla* (PH), *Sophora viciifolia* (SV), *Platycladus orientalis* (PO), *Armeniaca sibiriea* (AS) and *Rosa xanthina* (RX). These species were selected for their variety of seed morphological characteristics and their high dominance or occurrence in the hilly-gullied Loess Plateau region.

### Rainfall simulation experiments

Rainfall simulation experiments were carried out in the Rainfall Simulation Hall of the State Key Laboratory of Soil Erosion and Dryland Farming on the Loess Plateau. The rainfall simulation experiments were performed on slopes with gradients of 10°, 15°, 20° and 25° under rainfall intensities of 50 mm/h, 100 mm/h and 150 mm/h, respectively. Each simulated rainfall lasted 60 min. Three replications were designed for each experiment, and the three replications were arranged in different rainfall events. A total of 36 experiments were conducted.

The size of the experimental soil bin was 2 m × 0.5 m × 0.5 m (Length × Width × Height). The loess soil from An'sai County of the typical hilly-gulled Loess Plateau was used in the experiments, and the soil bulk density was controlled to 1.10-1.15 g/cm^3^. Before the rainfall simulation experiment, seeds dyed with safranin for easy identification were placed on the area of 100 cm-120 cm (from the top down bin) of soil surface. Two AS seeds, three RX seeds and five each of the other 14 species' seeds were placed on the soil surface. A total of 75 seeds were placed in each experiment.

### Data collection and analysis

The seeds were washed away from soil surface were counted to calculate the seed loss ratio. Differences in the values of seed loss rate from the different experiments were tested using one-way ANOVA analysis. Variables related to seed size, such as weight (M), length (L, longest axis), width (W, intermediate axis) and height (H, shortest axis) of these seeds were measured, and other variables such as the surface (S= L × W), volume (V= L × W × H), density (D = M/V) and ratio of S/M (Surface/Mass) were calculated. Seed shape was characterized by the Flatness Index (FI = (L +W)/2H). The Flatness Index ranges from a value of 1 for spherical seeds to greater values for plane or spindle shapes [16]. The features on seed surface, the presence of seed appendages(hairs, wings, awns, etc.) and the ability of seeds to segregate mucilage in contact with water were observed (Table 1). The effects of seed size and shape on seed losses were analyzed using regression procedures.

**Table 1:** Seed morphology characteristics of the 16 species

Species	Weight (mg)	Length (mm)	Width (mm)	Height (mm)	Surface (mm^2^)	Volume (mm^3^)	Density (mg/mm^3^)	Specific surface (mm^2^/mg)	FI	Appendix	Surface features
HA	0.29	2.32	1.32	0.55	3.07	1.69	0.17	10.48	3.31	pappus	dense depressed hair
BB	6.83	19.23	0.86	0.79	16.57	13.06	0.52	2.43	12.75	bur	rough, longitudinally angular
LD	2.14	3.44	2.00	0.94	6.89	6.48	0.33	3.22	2.89	none	dense pubes
SV	21.78	4.12	3.01	2.70	12.38	33.43	0.65	0.57	1.32	none	smooth
RP	20.22	4.84	3.49	1.78	16.90	30.06	0.67	0.84	2.34	none	smooth
RX	323.44	12.09	9.46	9.35	114.37	1069.88	0.30	0.35	1.15	none	smooth
CM	42.40	5.81	4.60	3.86	26.68	102.91	0.41	0.63	1.35	none*	smooth
AS	1108.79	19.42	14.80	10.19	287.41	2927.24	0.38	0.26	1.68	none	obviously reticulate lineate
BI	0.08	4.70	0.54	0.32	2.53	0.81	0.10	30.71	8.16	none	smooth, pubes
PS	4.82	7.53	1.69	0.84	12.70	10.69	0.45	2.63	5.48	villus	rough, small angulate
PO	13.41	5.25	3.17	2.45	16.64	40.85	0.33	1.24	1.72	none	smooth
PH	0.92	4.79	3.37	2.24	16.16	36.22	0.03	17.48	1.82	wing	rough, nettedvenation
DM	1.27	2.36	1.45	0.82	3.42	2.81	0.45	2.69	2.32	none	rough
CF	2.86	4.31	2.89	0.78	12.44	9.68	0.30	4.34	4.63	styles**	rough, dense short fuzz
RC	18.48	4.78	4.37	3.81	20.89	79.50	0.23	1.13	1.20	none	rough
DC	2.64	4.10	3.14	1.85	12.87	23.83	0.11	4.88	1.95	setaia	rough, obviously angulate

Species: HA - *Heteropappus altaicus*; BB - *Bidens bipinnata*; DM - *Dracocephalum moldavica*; CF - *Clematis florida*; LD - *Lespedeza davurica*; PS - *Periploca sepium*; DC - *Daucus carota*; BI - *Bothriochloa ischaemun*; RP - *Robinia psendoacacia*; RC - *Rubia cordifolia*; CM - *Cotoneaster multiflorus*; PH - *Patrinia heterophylla*, SV - *Sophora viciifolia*; PO - *Platycladus orientalis*; AS - *Armeniaca sibiriea*; RX - *Rosa xanthina*. * having mucilage secretion; **Plumose persistent styles

## Results

### Seed losses

The seed loss rate of the 16 species at the four slopes and at three simulated rainfall intensities is shown in Table 2. In the 50mm h^-1^ simulated rainfall, only one seed of LD on the 25° slope and one seed of BI on the 15° slope were lost out of just in one replication. When the rainfall intensity reached 100 mm/h, 10-13 species had seed loss on the four slopes, and the average seed loss rates of all species on the 10°, 15°, 20° and 25° slopes were 34.7%, 31.7%, 27.7% and 36.3% (P = 0.907), respectively. When the rainfall intensity was 150 mm/h, 13 species had seed loss on the four slopes with the average seed loss rate of 65.7%, 69.3%, 63.2% and 62.9% (P = 0.946), respectively. The range of seed loss rate among species was 0-86.7% and 0-100% at 100 mm/h and 150 mm/h, respectively. The seed loss obviously increased with rainfall intensity but did not obviously change with slope gradient, and the responses varied among the species.

**Table 2:** The seed loss rate the 16 species on the four slopes at the three simulated rainfall intensities

Species	Seed loss rate (%)											
	**50 mm h** ^**-1**^				**100 mm h** ^**-1**^				**150 mm h** ^**-1**^			
	**10°**	**15°**	**20°**	**25°**	**10°**	**15°**	**20°**	**25°**	**10°**	**15°**	**20°**	**25°**
HA	0.00	0.00	0.00	0.00	60.00	73.33	65.00	80.00	100.00	100.00	100.00	100.00
BB	0.00	0.00	0.00	0.00	20.00	0.00	5.00	10.00	45.00	60.00	24.00	25.00
DM	0.00	0.00	0.00	0.00	0.00	0.00	0.00	0.00	0.00	0.00	0.00	0.00
CF	0.00	0.00	0.00	0.00	10.00	13.33	10.00	0.00	50.00	75.00	56.00	60.00
LD	0.00	0.00	0.00	10.00	80.00	86.67	75.00	70.00	100.00	100.00	100.00	100.00
PS	0.00	0.00	0.00	0.00	70.00	73.33	70.00	60.00	100.00	100.00	100.00	100.00
DC	0.00	0.00	0.00	0.00	40.00	60.00	25.00	30.00	95.00	85.00	68.00	75.00
BI	0.00	10.00	0.00	0.00	70.00	66.67	80.00	50.00	100.00	100.00	96.00	90.00
RP	0.00	0.00	0.00	0.00	10.00	20.00	15.00	30.00	55.00	100.00	76.00	80.00
RC	0.00	0.00	0.00	0.00	30.00	0.00	0.00	0.00	65.00	75.00	64.00	60.00
CM	0.00	0.00	0.00	0.00	0.00	0.00	0.00	0.00	20.00	50.00	44.00	60.00
PH	0.00	0.00	0.00	0.00	30.00	33.33	20.00	20.00	75.00	70.00	56.00	60.00
AS	0.00	0.00	0.00	0.00	0.00	0.00	0.00	0.00	0.00	0.00	0.00	0.00
SV	0.00	0.00	0.00	0.00	60.00	73.33	25.00	70.00	90.00	80.00	80.00	70.00
PO	0.00	0.00	0.00	0.00	20.00	26.67	25.00	20.00	90.00	70.00	84.00	85.00
RX	0.00	0.00	0.00	0.00	0.00	0.00	0.00	0.00	0.00	0.00	0.00	0.00
Average	0.00	0.63	0.00	0.63	31.25	32.92	25.94	27.50	61.56	66.56	59.25	60.31

Species: HA - *Heteropappus altaicus*; BB - *Bidens bipinnata*; DM - *Dracocephalum moldavica*; CF - *Clematis florida*; LD - *Lespedeza davurica*; PS - *Periploca sepium*; DC - *Daucus carota*; BI - *Bothriochloa ischaemun*; RP - *Robinia psendoacacia*; RC - *Rubia cordifolia*; CM - *Cotoneaster multiflorus*; PH - *Patrinia heterophylla*, SV - *Sophora viciifolia*; PO - *Platycladus orientalis*; AS - *Armeniaca sibiriea*; RX - *Rosa xanthina*

### Seed loss vs. seed morphology characteristics

The correlation analysis showed that there were negative relationships between seed loss rate and seed length, width, height, surface, volume, density and weight, respectively. There were positive relationships between seed loss rate and specific surface and FI, respectively. At 150 mm/h rainfall, the negative correlation between seed loss rate and seed height and width reached a 0.01 significance level, while the seed weight, length, surface and volume reached a 0.05 significance level. However, the relationships between seed loss rate and density, specific surface and FI were not significant, respectively. While 100 mm/h rainfall, only the negative relationship between seed loss rate and seed width was significant at 0.05 level (Table 3).

**Table 3:** Seed size and shape in correlation with seed loss rate

Rainfall	Weight	Length	Width	Height	Surface	Volume	Density	Specific surface	FI
	**(mg)**	**(mm)**	**(mm)**	**(mm)**	**(mm** ^**2**^ **)**	**(mm** ^**3**^ **)**	**(mg/mm** ^**3**^ **)**	**(mm** ^**2**^ **/mg)**	
100 mm/h	-0.341	-0.431	-0.502*	-0.493	-0.389	-0.356	-0.184	0.428	0.105
150 mm/h	-0.584*	-0.544*	-0.644**	-0.667**	-0.616*	-0.607*	-0.184	0.365	0.132

Seed loss rate is an average of the four slopes under the same rainfall intensity. *a = 0.05; **a = 0.01; n = 16

However, the seed size or shape cannot qualitatively explain the variation of seed loss among species. For the seeds of different species, their specific morphological characteristics affecting seed loss were quite different. Species AS and RX, with a seed weight of more than 300 mg, did not have loss in any of the experimental conditions. The DM seeds, which are small and have a rough surface, can form mucilage when absorb water and not easily be washed away, and they had no seed loss in any of the experiments. The seeds of SV and PO, which have a smooth surface and spherical shape, had seed loss rates of 80.0% and 82.3% at 150 mm/h rainfall. The seeds of HA, DC and BI, which have very small weight and density, had higher seed loss rates of 69.6%, 38.6% and 66.7% at 100 mm/h rainfall, and 100%, 80.6% and 96.5% at 150 mm/h rainfall, respectively. LD seeds, which have a smooth surface, had seed loss rates of 77.9% and 100% at 100 mm/h and 150 mm/h rainfall, respectively. PS seeds, which are obround and have hairs at the top that fall off when meeting water, had high seed loss rates of 68.3% and 100% at 100 mm/h and 150 mm/h rainfall, respectively. RP seeds, which have a smooth surface and a relatively mid-level weight and density, had seed loss rates of 18.8% and 77.8% at 100 mm/h and 150 mm/h rainfall, respectively. RC seeds, which have a bigger volume, weight and a rough surface, had lower seed loss rates of 30.0% and 66.0% at 100 mm/h and 150 mm/h rainfall, respectively. Seeds from PH and CF, which have a rough surface and flat shape, had lower loss rates of 25.8% and 11.1% at 100 mm/h rainfall, and 65.3% and 60.3% at 150 mm/h rainfall. The CM fruit, which is close to spherical but has a bigger volume and weight, only had low losses at 150 mm/h rainfall with a rate of 43.5%. The BB seed is a long achene with 4 corners and 3 or 4 thorns with persistent barbed seta, and it only had low losses at 150 mm/h rainfall with a rate of 38.5% (Tables 1 and 2).

## Discussion

### Effect of seed morphology features on seed loss

There was a wide variation in seed losses between species, which could not be quantitatively explained by seed weight, size or shape indexes. Other features, such as mucilage secretion and the presence of appendices like as hairs or wings, may explain the differences in seed losses at the species level [9]. Seeds have specific mechanisms to avoid remobilization by water erosion, which may obscure the relationships between seed removal and seed quantitative indexes such as size or weight [9]. Combined with our pre-experiment, seed loss of some species are mainly affected by seed weight. For example, the seeds of RX, AS, *Malus baccata, Ziziphus jujuba*, Chinese date, *Glycine max* and *Zea mays* with weights over 190 mg were able to avoid loss, while the light seeds of HA, DC and BI had high loss rates. Some species are mainly due to seed shape, such as the elongated seed of BB and *Stipa grandis* had low loss rate and the winged fruit of *Acer ginnala* could avoid loss; while the spherical-shaped seed of SV had higher seed loss rate. Some are affected by seed surface structure, such as LD seeds, which have a smooth surface, were easily washed away. While DM seeds, having the ability to secrete mucilage under moist conditions, do not show loss in any of the experiment events. In short, seed removal effected by its specific morphology characteristics, and the seeds form different species have various responses to water erosion.

### Effect of seed loss on vegetation development on eroded slopes

Based on 174 samples in the hilly-gullied region on the Loess Plateau, the TWINSPAN analysis showed the main plant communities include the dominant species *Artemisia scoparia*, *Leymus scalinus*, *Lespedeza davurica*, *Stipa bungeana*, *Artemisia gmelinii*, *Artemisia giraldii* and *Bothriochloa ischaemun* in different combinations, and these species have higher cover and frequency [17]. For these seven species, We found that the seeds of *Artemisia scoparia*, *Artemisia gmelinii* and *Artemisia Giralaii* also have mucilage secretion and not easily to be washed away by water erosion. The mucilage mass can also facilitate seed germination and seedling development [18]. This may be one of the reasons these three *Artemisia* plants are the dominant species on the Loess Plateau. *A. scoparia* is the pioneer plant in the early restoration of abandoned land which undergoing severe soil erosion. *A. scoparia* has high seed yield (0.2-5.3 × 10^5^ seeds m^-2^) with very small size (0.02 mg), and is easily propagated by wind and buried into soils. In the natural vegetation of the hilly-gully environment, the soil seed bank was dominated by *A. scoparia* with a high seed density of 1 000-17 000 seed m^-2^ in a 0-10-cm soil layer [19]. While *Leymus scalinus* has a low ripening rate and regenerated by rhizome with a film distribution. The seeds from *S. bungeana*, having bur with elongated shape, had low seed loss rate. *S. bungeana* is the main dominant Gramineae species in the area with a higher cover (20-70%) and higher occurrence. *S. bungeana*, *S. bungeana* + LD, *A. gmelinii* + *S. bungeana*, and *A. giraldii* + *S. bungeana* are the common communities in the area. Apart from seminal propagation, another special mode of reproduction for *S. bungeana* is by the bulbiet falling off the parent plant to develop a new plant [20]. The seeds of LD and BI had higher seed loss rate. In the eroded slopes, LD and BI have soil seed banks with a density of 50-300 seeds m^-2^; however, the seed density can reach 300-1,000 seeds m^-2^ in silted areas [19]. LD also has a large root system and strong shooting ability. While BI is the dominant species of the perennial herb and subshrub community in the middle and late stages of vegetation restoration. The seeds of BI are small, and easily washed away by overland flow. Moreover, the viability of BI seedlings is low; BI seedlings are weak due to the competition among early subshrub species for limited environmental resources, and sporadically and filmily distributed in the hilly-gullied Loess plateau. Short rhizome generation is used by BI as an ecological adaption strategy. It was observed that BI mainly relies on clonal propagation to achieve population renewal in the natural meadow [21]. Thus, vegetative propagation also plays a very important role in vegetation development in the Loess Plateau region.

For the other species, DM can be found in eroded slopes, such as early abandoned lands and new roadsides; *Stipa grandis* also had no seed loss in our pre-experiment and can be found in steep slopes; PS can form sparse communities on lower slopes because the PS seeds are easy to transfer by water erosion; SV is shrub with a partial and scattered distribution in the middle and later stages of vegetation succession on the Loess Plateau, and can be found in lower inter-gullies and in the gullies. It seems that seed loss has effect on vegetation distribution. Moreover, in the eroded slopes in the Zhifanggou catchment in the typical hill-gully area on the Loess Plateau, the density of soil seed bank in the lower silted places is significantly larger than that in the gap between vegetation; and the vegetation tussock and the silted area can hold up the seed of most species, while the gap vegetation loses the seed of many species and is dominated by the pioneer plant *Artemisia scoparia* [19].

Above all, seed removal is likely to affect seed redistribution and deposition and, consequently, vegetation development and distribution, while seed removal effected by seed morphology characteristics. Thus, the species composition of stable communities on eroded slopes may provide references for species selection in vegetation rehabilitation for soil erosion control. However, the seed removal in this study was conducted on bare loess slopes, and the seed removal under natural conditions in the Loess Plateau region needs to be further investigated.
